# Supplementary material for: A “one-size-fits-most” walking recognition method for smartphones, smartwatches, and wearable accelerometers
Source: NPJ Digit Med. 2023 Feb 23;6:29. doi: 10.1038/s41746-022-00745-z (PMC9950089; doi:10.1038/s41746-022-00745-z)
Supplement: Supplementary file 1 — Supplementary Material [file 41746_2022_745_MOESM1_ESM.docx]

**Supplementary Table 1**. Selected methods for walking recognition using body-worn device data (for further read on activity recognition methods using smartphones, see ^1^).

| **Sensing device** | **Device location** | **Sensors involved** | **Population** | **Validation setting** | **Investigated activities** | **Main concept** | **Reference** |
| --- | --- | --- | --- | --- | --- | --- | --- |
| Smartphone | Front and back pants pocket | Accelerometer and gyroscope | N=4 (2 males)  Age: 27-54 y | Controlled | Walking, ascending stairs, descending stairs, sitting, standing | Tri-axial accelerometer and gyroscope data are used as activity templates. Walking is identified when the distance between new data fragment and activity template exceeds the threshold. | ^2^ |
| Smartphone | Front pants pocket | Gyroscope | N=8 (5 males), Age: 23-26 y | Controlled | Walking, stair climbing, standing, typing | Fast Fourier transform is computed over most sensitive axis. Walking is identified when the average area under the spectrum within predefined range exceeds area under the spectrum below with range. | ^3^ |
| Smartphone | Hand, pants pocket, backpack | Accelerometer and gyroscope | N=77 | Controlled | Walking, non-walking | Authors investigate several classification approaches, e.g., using 21 time- and frequency domain features combined with various machine learning techniques, activity-templates, and deep learning. Deep learning provides the highest classification accuracy. | ^4^ |
| Smartphone | Pants pocket | Accelerometer | N=32 (16 males), age: ~25 y | Controlled | Walking | Tri-axial accelerometer data is filtered using a low-pass filter. Activity templates, time-domain features, and frequency-domain features are extracted for multiple machine learning classifiers. Walking is identified using voting method. | ^5^ |
| Wearable accelerometer | Wrist | Accelerometer | N=20 (15 males), age: 26.8±3.6 y | Controlled | Normal walking, fast walking, random hand movement | Vector magnitude is segmented using adaptive window based on local maxima that indicate walking steps. Several time-domain features are extracted for each segment. Walking is identified using threshold on anomaly detection score. | ^6^ |
| Wearable accelerometer | Wrist | Accelerometer | Dataset 1: N=5 (4 males); age: 29.4±2.1 y  Dataset 2: N=10 (8 males), age: 27.2±3.1 y  Dataset 3: N=3 | Controlled | Dataset 1: walking, cycling, running, lying, sitting, standing, kneeling, bending, body transitions  Dataset 2: treadmill walking, treadmill running, stationary bike cycling, sitting, standing, lying, bending, kneeling, body transitions  Dataset 3: walking, standing, sitting, lying | Segmented tri-axial data is filtered using low-pass and band-pass filters. Several time-domain features are extracted. Feature selection is performed to identify most informative features. Machine learning (Random Forest) classifier is used to distinguish walking from other activities in the dataset. | ^7^ |
| Wearable accelerometer | Waist | Accelerometer | Dataset 1: N=20 (10 males); healthy; Age: 68.5±7.4 y  Dataset 2: N=32 (17 males); PD; 67.3±6.6 y | Controlled | Normal walking, slow walking, fast walking, walking while carrying a tray | Walking is identified when the absolute resultant of the three axes data exceeds a threshold. | ^8^ |
| Wearable accelerometer | Hip | Accelerometer | N=49 (25 males)  Age: 78 y (IQ: 74-82 y) | Controlled | Fast walking, dressing, shopping, chair stands | Raw tri-axial data are transformed into vector magnitude; vector magnitude is transformed into frequency-domain using Fast Fourier transform; walking is identified when the highest ratio between (a) and (b) exceeds the threshold, where (a) area under the spectrum at baseline frequency and its harmonics for each baseline frequency bin, and (b) is area under the spectrum for all frequencies. | ^9^ |
| Wearable accelerometer | Lower back | Accelerometer | N=10 (20-33), age: 27.5±4.7 y, healthy | Free-living | Walking in various context, other non-walking activities | Tri-axial data are filtered out from high-frequency noise (17 Hz and above) and transformed to fixed (horizontal-vertical) coordinate system. A moving standard deviation and vertical acceleration detects upright movement. Micro-gait events (initial contact & final contact) detected using continuous wavelet transform are used to identify walking. | ^10^ |
| Wearable accelerometer | Wrist, ankle | Accelerometer | (1) N=33 (11 males), age: 18-75 y  (2) N=20 (12 males), age: 13±1.3 y | Controlled | Various types of walking (while carrying item, on treadmill, etc.), stair climbing, cycling, playing basketball, tennis, and soccer, painting, house cleaning, sitting, lying, sitting | Vector magnitude is preprocessed using low-pass filter (15 Hz). Segmented data are used to extract several time- and frequency-domain. New features based on signal fragmentation are used. Machine learning (SVM) technique is used to distinguish between ambulation, cycling, sedentary, and activities outside of these categories. | ^11^ |
| Wearable accelerometer | Hip, wrist | Accelerometer | N=40 (0 males), age: 55.2±15.3 y, BMI: 32.0±3.7 | Free-living | Walking/running, sitting, standing, riding in a vehicle | Raw (unfiltered) tri-axial acceleration and its vector magnitude data are used to extract 41 time- and frequency-domain features. Feature vectors are used in Random Forest classifier to predict performed activities. Hidden Markov model is used to smooth predictions over time. | ^12^ |
| Wearable accelerometer | Wrist, ankle | Accelerometer | Dataset 1: N=33 (11 males), age: 18-75 y  Dataset 2: N=35 (14 males), age: 65-80 y  Dataset 3: N=15 (6 males), age: 65-78 y | Controlled (1-2), free-living (3) | Ambulation, cycling, sedentary, and activities outside of these categories | Tri-axial data are used to compute 13 or 77 time- and frequency-domain features. Conditional Random Fields is used for recognition of ambulation. | ^13^ |
| Wearable accelerometer | Ankle, hip, wrist | Accelerometer | N=35 (14 males), age: 70.8±4.9 y | Free-living | Locomotion (slow walk, self-paced 400m walk, carrying groceries), household (dusting, gardening, vacuuming, self-care, laundry, organizing the room), sedentary (lying down, sitting, crossword puzzles, playing cards), standing (stationary), recreational activities (tai chi, simulated bowling) | Tri-axial data is used to extract several time- and frequency-domain features. Support vector machines and Random Forest methods were used to classify locomotion activities. | ^14^ |
| Wearable accelerometer | Hip | Accelerometer | N=20, older | Controlled | Normal walking, fast walking, dealing cards, standing from a chair, shopping, handwriting, vacuuming, folding towels, dressing, kneading, washing dishes, standing, lying. | Tri-axial data is transformed into vector magnitude and normalized using linear transformation. Extracted activity templates are used for activity classification based on the minimum distance from an activity pattern. | ^15^ |
| Wearable accelerometer | Wrist, hip | Accelerometer | N=60 (23 males), age: 40-65 y | Controlled | Lying, standing, seated computer work, treadmill walk, ascending and descending stairs, normal walk, washing windows, washing up, shelf stacking, sweeping, running, | Segmented data is transformed into vector magnitude and used to extract several time- and frequency-domain features. Support vector machines is used to select most informative features. Decision tree is used to distinguish between sedentary, household, walking, and running activities. | ^16^ |

**Supplementary Table 2.** Activity groups used in our study. Note that activity names in column “Included activities” were given by researchers collecting datasets.

| Activity group | Included activities |
| --- | --- |
| Walking |  |
| Normal walking | Walking, fast walking, slow walking |
| Non-walking |  |
| Stationary | Lying, sitting, standing, standing in the elevator |
| Desk work & TV | Watching TV, working on the computer, typing, handwriting |
| Eating | Eating, eating pasta, eating sandwich, eating soup, eating meat |
| Drinking | Pouring, drinking, drinking coffee |
| Motorized | Car driving, travelling in the car, motorcycle, and rickshaw |
| Other | Moving in the elevator, smoking, giving a talk, using telephone, coughing, sneezing, body transitions, clapping |

**Supplementary Table 3. Supplementary bias model estimates**. Coefficient estimates, standard errors, and 95% confidence intervals without Bonferroni correction and 99.5% with Bonferroni correction. **a.** *StandardReg* model. **b.** *MixedReg* model. In **a.** and **b.**, the covariate age in years was standardized by centering with the mean (28.7 y) and dividing by the standard deviation (12.0 y). BMI was also standardized by centering with the mean (22.9 kg/m2) and dividing by the standard deviation (3.9 kg/m2). Sex, environmental condition, and sensor location are incorporated using indicator variables. The reference category for sex is female, the reference category for environment is the controlled setting, and the reference category for sensor location is the arm, and the reference category for device is wearable. Compared to Table 3, this model includes information about the data collection device (wearable, smartphone) and original data sampling frequency, but removes the study acronyms.

|  | **Estimate** | **Standard error** | **95% Confidence Interval**  (without Bonferroni correction) | **99.5% Confidence Interval**  (with Bonferroni correction) |
| --- | --- | --- | --- | --- |
| a. *StandardReg* | | | | |
| Intercept | 0.8990 | 0.0232 | (0.8535, 0.9445) | (0.8330, 0.9650) |
| Age | 0.0032 | 0.0043 | (-0.0053, 0.0117) | (-0.0091, 0.0155) |
| BMI | 0.0068 | 0.0040 | (-0.0011, 0.0147) | (-0.0046, 0.0182) |
| Sex |  |  |  |  |
| Male | 0.0066 | 0.0089 | (-0.0109, 0.0242) | (-0.0188, 0.0320) |
| Measurement condition |  |  |  |  |
| Free-living | -0.0188 | 0.0163 | (-0.0508, 0.0133) | (-0.0652, 0.0277) |
| Sensor location |  |  |  |  |
| Thigh | 0.0123 | 0.0217 | (-0.0303, 0.0549) | (-0.0495, 0.0740) |
| Waist | 0.0564 | 0.0224 | (0.0123, 0.1004) | (-0.0074, 0.1202) |
| Chest | 0.0726 | 0.0256 | (0.0223, 0.1229) | (-0.0003, 0.1455) |
| Wrist | 0.0299 | 0.0231 | (-0.0155, 0.0753) | (-0.0358, 0.0957) |
| Unspecified | 0.0295 | 0.0266 | (-0.0227, 0.0817) | (-0.0461, 0.1052) |
| Device |  |  |  |  |
| Smartphone | -0.0004 | 0.0098 | (-0.0196, 0.0188) | (-0.0283, 0.0275) |
| Sampling frequency | -0.0043 | 0.0059 | (-0.0159, 0.0073) | (-0.0212, 0.0125) |
| b. *MixedReg* | | | | |
| Intercept | 0.9235 | 0.0204 | (0.8833, 0.9643) | (0.8648, 0.9823) |
| Age | 0.0057 | 0.0050 | (-0.0039, 0.0155) | (-0.0086, 0.0199) |
| BMI | 0.0052 | 0.0049 | (-0.0043, 0.0149) | (-0.0089, 0.0197) |
| Sex |  |  |  |  |
| Male | 0.0101 | 0.0108 | (-0.0112, 0.0313) | (-0.0218, 0.0416) |
| Measurement condition |  |  |  |  |
| Free-living | -0.0157 | 0.0170 | (-0.0491, 0.0183) | (-0.0633, 0.0334) |
| Sensor location |  |  |  |  |
| Thigh | -0.0142 | 0.0180 | (-0.0492, 0.0214) | (-0.0654, 0.0384) |
| Waist | 0.0216 | 0.0180 | (-0.0142, 0.0576) | (-0.0283, 0.0732) |
| Chest | 0.0306 | 0.0207 | (-0.0109, 0.0718) | (-0.0288, 0.0890) |
| Wrist | -0.0026 | 0.0184 | (-0.0391, 0.0337) | (-0.0563, 0.0504) |
| Unspecified | -0.0128 | 0.0226 | (-0.0571, 0.0312) | (-0.0815, 0.0536) |
| Device |  |  |  |  |
| Smartphone | 0.0041 | 0.0112 | (-0.0180, 0.0260) | (-0.0280, 0.0351) |
| Sampling frequency | -0.0065 | 0.0071 | (-0.0203, 0.0075) | (-0.0269, 0.0129) |

**Supplementary Table 4.** Walking classification accuracy over data with uniform sampling rate equal to 15 Hz.

| **Locations typical to** | **Smartphone** | | | | | **Smartwatch** |
| --- | --- | --- | --- | --- | --- | --- |
|  | **Thigh** | **Waist** | **Chest** | **Arm** | **Unspecified** | **Wrist** |
| Walking |  |  |  |  |  |  |
| Normal walking | 0.95 (0.93,0.96), 459 | 0.96 (0.95,0.97), 538 | 0.96 (0.93,0.98), 110 | 0.93 (0.9,0.97), 60 | 0.96 (0.95,0.97), 273 | 0.94 (0.93,0.96), 352 |
| Stair climbing |  |  |  |  |  |  |
| Ascending stairs | 0.84 (0.82,0.87), 361 | 0.86 (0.84,0.89), 396 | 0.93 (0.9,0.97), 74 | 0.83 (0.76,0.89), 60 | 0.91 (0.86,0.95), 69 | 0.75 (0.71,0.8), 222 |
| Descending stairs | 0.86 (0.83,0.88), 364 | 0.86 (0.84,0.89), 392 | 0.79 (0.72,0.85), 74 | 0.81 (0.74,0.89), 62 | 0.84 (0.78,0.91), 70 | 0.74 (0.69,0.78), 213 |
| Treadmill |  |  |  |  |  |  |
| 1 mph | 0.18 (0.11,0.25), 31 | 0.05 (0.03,0.08), 31 | - | - | - | 0 (0,0), 31 |
| 2 mph | 0.83 (0.72,0.94), 30 | 0.81 (0.69,0.92), 30 | - | - | - | 0.06 (0,0.11), 30 |
| 3 mph | 0.99 (0.99,1), 29 | 0.99 (0.99,1), 29 | - | - | - | 0.93 (0.87,0.98), 29 |
| 3.5 mph | 0.99 (0.98,1), 28 | 0.99 (0.98,1), 28 | - | - | - | 0.81 (0.7,0.92), 28 |
| Other walking | 0.87 (0.8,0.94), 17 | 0.77 (0.66,0.89), 17 | 0.85 (0.78,0.93), 17 | - | - | 0.56 (0.4,0.73), 17 |
| Non-walking |  |  |  |  |  |  |
| Stationary & TV | 0.99 (0.99,1), 401 | 1 (1,1), 380 | 0.99 (0.99,1), 89 | 0.99 (0.96,1.01), 60 | 1 (0.99,1), 133 | 0.99 (0.98,0.99), 257 |
| Desk work | 1 (1,1), 153 | 1 (1,1), 33 | - | - | 0.98 (0.98,0.99), 35 | 1 (1,1), 154 |
| Eating | 1 (0.99,1), 212 | - | - | - | 0.97 (0.96,0.98), 57 | 0.99 (0.98,1), 214 |
| Drinking | 0.99 (0.98,1.01), 61 | - | - | - | - | 0.99 (0.99,1), 81 |
| Motorized transport | - | 1 (1,1), 32 | - | - | 0.91 (0.9,0.93), 117 | 0.99 (0.99,1), 32 |
| Household |  |  |  |  |  |  |
| Sweeping | 0.94 (0.91,0.97), 34 | 0.94 (0.92,0.96), 53 | 0.87 (0.84,0.91), 19 | - | 0.98 (0.96,1.01), 2 | 0.54 (0.48,0.59), 53 |
| Vacuuming | - | 0.98 (0.96,0.99), 19 | 0.99 (0.98,1), 19 | - | - | 0.87 (0.77,0.97), 19 |
| Folding clothes | 0.97 (0.95,1), 51 | - | - | - | - | 0.68 (0.63,0.73), 51 |
| Washing dishes | - | 1 (1,1), 19 | 1 (1,1), 19 | - | 1 (0.99,1), 5 | 0.96 (0.92,1), 19 |
| Grooming | - | - | - | - | 0.93 (0.86,1.01), 15 | - |
| Dressing | - | - | - | - | 0.91 (0.74,1.07), 8 | - |
| Cooking | - | - | - | - | 0.98 (0.96,1), 12 | - |
| Filling shelves | 0.95 (0.92,0.97), 32 | 0.98 (0.97,1), 32 | - | - | - | 0.7 (0.64,0.76), 32 |
| Personal hygiene |  |  |  |  |  |  |
| Combing hair | - | - | - | - | - | 0.65 (0.4,0.9), 5 |
| Brushing teeth | 1 (1,1), 51 | - | - | - | - | 1 (0.99,1), 54 |
| Sports |  |  |  |  |  |  |
| Running | 0.96 (0.95,0.97), 432 | 0.98 (0.96,0.99), 421 | 0.96 (0.93,0.99), 114 | 0.98 (0.96,0.99), 61 | 0.92 (0.89,0.95), 126 | 0.98 (0.96,0.99), 264 |
| Cycling | 0.96 (0.94,0.98), 62 | 0.95 (0.92,0.98), 90 | 0.98 (0.95,1), 48 | 0.98 (0.96,1), 10 | 0.85 (0.77,0.94), 23 | 0.99 (0.99,1), 110 |
| Jumping | 0.13 (0.1,0.16), 326 | 0.18 (0.14,0.21), 354 | 0.13 (0.07,0.18), 85 | 0.13 (0.06,0.2), 50 | 0.14 (0.06,0.22), 61 | 0.19 (0.14,0.24), 176 |
| Other |  |  |  |  |  |  |
| Hand clapping | 0.99 (0.97,1), 51 | - | - | - | - | 0.92 (0.88,0.97), 51 |
| Smoking | 1 (1,1), 10 | - | - | - | - | 1 (1,1), 10 |
| Giving a talk | 1 (1,1), 10 | - | - | - | - | 0.94 (0.9,0.98), 10 |
| Body transitions | 0.95 (0.93,0.98), 108 | 0.98 (0.97,0.99), 85 | 0.87 (0.81,0.93), 17 | - | - | 1 (0.99,1), 29 |
| Coughing | 1 (1,1), 17 | 1 (1,1), 17 | 1 (1,1), 17 | - | - | 1 (1,1), 17 |

**Supplementary Table 5.** Walking classification accuracy achieved using modified tuning parameters of amplitude threshold and step frequency range ($A$ = 0.1 g, $f_{w}$ = [0.9 Hz, 2.3 Hz]). The accuracy is provided as mean (95% CI), sample size. For walking activities, the metric indicates sensitivity; for non-walking activities, the metric indicates specificity.

| **Locations typical to** | **Smartphone** | | | | | **Smartwatch** |
| --- | --- | --- | --- | --- | --- | --- |
|  | **Thigh** | **Waist** | **Chest** | **Arm** | **Unspecified** | **Wrist** |
| Walking |  |  |  |  |  |  |
| Normal walking | 0.96 (0.95,0.97), 459 | 0.98 (0.97,0.98), 538 | 0.99 (0.98,0.99), 110 | 0.96 (0.93,0.99), 60 | 0.95 (0.94,0.96), 273 | 0.95 (0.94,0.97), 352 |
| Stair climbing |  |  |  |  |  |  |
| Ascending stairs | 0.91 (0.89,0.93), 361 | 0.92 (0.9,0.94), 396 | 0.98 (0.96,1), 74 | 0.97 (0.94,0.99), 60 | 0.96 (0.92,0.99), 69 | 0.8 (0.76,0.85), 222 |
| Descending stairs | 0.9 (0.88,0.92), 364 | 0.89 (0.87,0.91), 392 | 0.81 (0.74,0.87), 74 | 0.84 (0.76,0.92), 62 | 0.86 (0.79,0.92), 70 | 0.76 (0.72,0.81), 213 |
| Treadmill |  |  |  |  |  |  |
| 1 mph | 0.8 (0.73,0.86), 31 | 0.61 (0.52,0.69), 31 | - | - | - | 0 (0,0), 31 |
| 2 mph | 0.95 (0.92,0.98), 30 | 0.94 (0.88,0.99), 30 | - | - | - | 0.08 (0.03,0.12), 30 |
| 3 mph | 0.98 (0.97,0.99), 29 | 0.99 (0.99,1), 29 | - | - | - | 0.94 (0.89,0.98), 29 |
| 3.5 mph | 0.98 (0.96,1), 28 | 0.99 (0.98,1), 28 | - | - | - | 0.87 (0.8,0.93), 28 |
| Other walking | 0.99 (0.99,1), 17 | 0.99 (0.97,1), 17 | 0.94 (0.91,0.98), 17 | - | - | 0.71 (0.55,0.87), 17 |
| Non-walking |  |  |  |  |  |  |
| Stationary & TV | 0.98 (0.98,0.99), 401 | 0.99 (0.99,0.99), 380 | 0.99 (0.98,1), 89 | 0.99 (0.98,0.99), 60 | 0.99 (0.98,1), 133 | 0.97 (0.96,0.98), 257 |
| Desk work | 0.99 (0.99,1), 153 | 1 (1,1), 33 | - | - | 0.96 (0.95,0.98), 35 | 0.99 (0.99,1), 154 |
| Eating | 0.98 (0.97,0.99), 212 | - | - | - | 0.94 (0.92,0.95), 57 | 0.9 (0.87,0.92), 214 |
| Drinking | 0.98 (0.96,1.01), 61 | - | - | - | - | 0.98 (0.96,1), 81 |
| Motorized transport | - | 0.87 (0.86,0.89), 32 | - | - | 0.68 (0.65,0.7), 117 | 0.89 (0.87,0.91), 32 |
| Household |  |  |  |  |  |  |
| Sweeping | 0.67 (0.59,0.75), 34 | 0.68 (0.63,0.74), 53 | 0.41 (0.34,0.48), 19 | - | 0.97 (0.91,1.03), 2 | 0.28 (0.23,0.33), 53 |
| Vacuuming | - | 0.6 (0.51,0.69), 19 | 0.56 (0.45,0.67), 19 | - | - | 0.6 (0.47,0.72), 19 |
| Folding clothes | 0.89 (0.83,0.95), 51 | - | - | - | - | 0.48 (0.42,0.55), 51 |
| Washing dishes | - | 1 (1,1), 19 | 0.99 (0.99,1), 19 | - | 0.97 (0.93,1.02), 5 | 0.78 (0.72,0.85), 19 |
| Grooming | - | - | - | - | 0.87 (0.77,0.97), 15 | - |
| Dressing | - | - | - | - | 0.84 (0.63,1.05), 8 | - |
| Cooking | - | - | - | - | 0.93 (0.89,0.98), 12 | - |
| Filling shelves | 0.69 (0.62,0.75), 32 | 0.75 (0.71,0.8), 32 | - | - | - | 0.4 (0.33,0.48), 32 |
| Personal hygiene |  |  |  |  |  |  |
| Combing hair | - | - | - | - | - | 0.41 (0.07,0.74), 5 |
| Brushing teeth | 0.99 (0.97,1), 51 | - | - | - | - | 0.93 (0.89,0.97), 54 |
| Sports |  |  |  |  |  |  |
| Running | 0.91 (0.89,0.93), 431 | 0.97 (0.96,0.98), 430 | 0.95 (0.91,0.98), 114 | 0.94 (0.91,0.98), 60 | 0.86 (0.82,0.91), 126 | 0.97 (0.95,0.98), 264 |
| Cycling | 0.56 (0.47,0.65), 62 | 0.48 (0.41,0.56), 90 | 0.77 (0.69,0.85), 48 | 0.79 (0.64,0.93), 10 | 0.66 (0.57,0.75), 23 | 0.95 (0.94,0.97), 110 |
| Jumping | 0.1 (0.08,0.13), 326 | 0.16 (0.13,0.19), 354 | 0.12 (0.06,0.18), 85 | 0.07 (0.03,0.11), 50 | 0.06 (0.01,0.11), 61 | 0.15 (0.11,0.19), 176 |
| Other |  |  |  |  |  |  |
| Hand clapping | 0.96 (0.93,0.99), 51 | - | - | - | - | 0.89 (0.84,0.94), 51 |
| Smoking | 0.99 (0.97,1), 10 | - | - | - | - | 0.97 (0.94,1), 10 |
| Giving a talk | 0.99 (0.98,1), 10 | - | - | - | - | 0.68 (0.59,0.77), 10 |
| Body transitions | 0.76 (0.7,0.82), 108 | 0.76 (0.71,0.8), 85 | 0.56 (0.46,0.66), 17 | - | - | 0.97 (0.93,1.01), 29 |
| Coughing | 1 (1,1), 17 | 1 (1,1), 17 | 1 (1,1), 17 | - | - | 1 (1,1), 17 |


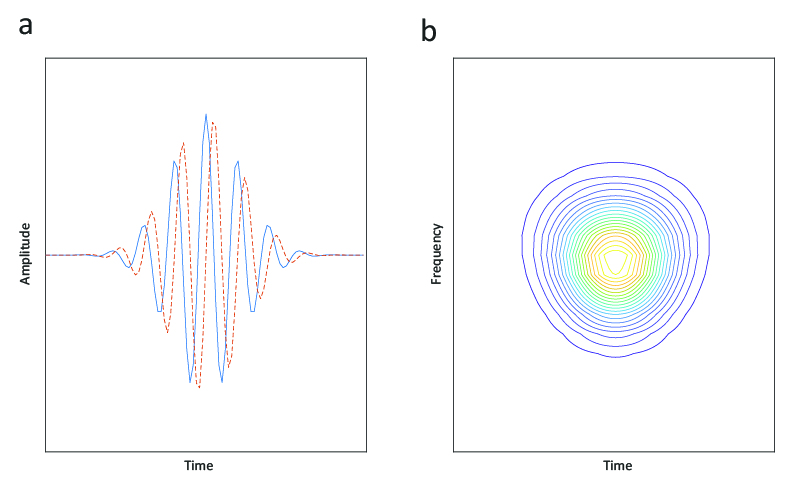


**Supplementary Figure 1.** **A Mother wavelet, Morse, used in the walking recognition method.** a. The wavelet’s real part is displayed with the blue solid line; the imaginary part is displayed with the red dashed line. b. The wavelet has sampling frequency equal to 10 Hz and central frequency equal to 1 Hz. Its symmetry coefficient, $\gamma$, is equal to 3, while time-bandwidth product, $P^{2}$, is equal to 60, which provide symmetrical coefficients’ spread in time and frequency domains.


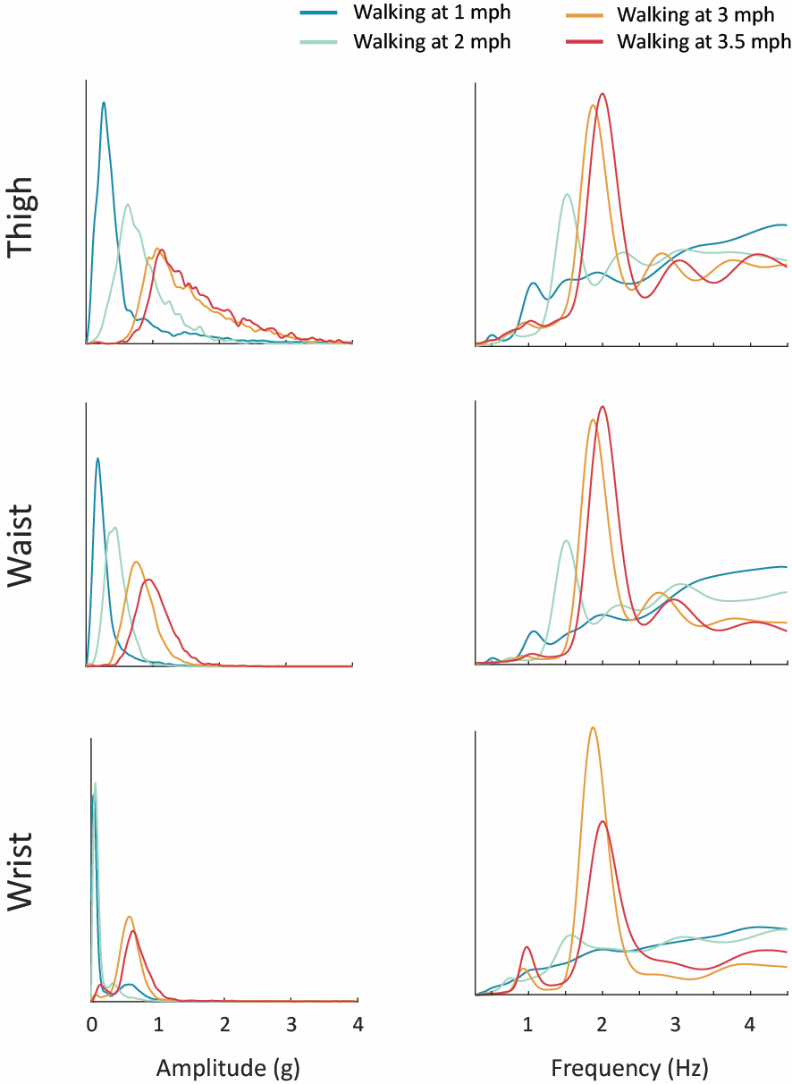


**Supplementary Figure 2. Amplitude and frequency distributions of treadmill walking at various speed and various sensor body locations observed in *SPADES* dataset.** Walking at 2 mph, 3 mph, and 3.5 mph demonstrate a distinct peak associated with step frequency around 1.50Hz, 1.84 Hz, and 1.96 Hz, respectively. The step frequency is less apparent at 1 mph (1.06 Hz), additionally reflecting in a very small acceleration amplitude (peaks at 0.32 g, 0.22 g, and 0.08g, for thigh, waist, and wrist, respectively). We hypothesize that during slow walking at 1 mph and 2 mph study participants were holding the treadmill rail with damped the amplitude of oscillations and effectively diminished step frequency information from the time-frequency projection. Distributions were calculated in a similar way to ones displayed in Figure 4.

**References**

1. Straczkiewicz, M., James, P. & Onnela, J.-P. A systematic review of smartphone-based human activity recognition methods for health research. *npj Digit. Med.* **4**, 148 (2021).

2. Huang, E. J. & Onnela, J.-P. Augmented Movelet Method for Activity Classification Using Smartphone Gyroscope and Accelerometer Data. *Sensors* **20**, (2020).

3. Kang, X., Huang, B. & Qi, G. A Novel Walking Detection and Step Counting Algorithm Using Unconstrained Smartphones. *Sensors (Basel).* **18**, (2018).

4. Casado, F. E. *et al.* Walking Recognition in Mobile Devices. *Sensors*  vol. 20 (2020).

5. Huan, Z., Chen, X., Lv, S. & Geng, H. Gait Recognition of Acceleration Sensor for Smart Phone Based on Multiple Classifier Fusion. *Math. Probl. Eng.* **2019**, 6471532 (2019).

6. Cola, G., Avvenuti, M., Musso, F. & Vecchio, A. Personalized gait detection using a wrist-worn accelerometer. in *2017 IEEE 14th International Conference on Wearable and Implantable Body Sensor Networks (BSN)* 173–177 (2017). doi:10.1109/BSN.2017.7936035.

7. Gjoreski, M., Gjoreski, H., Lustrek, M. & Gams, M. How Accurately Can Your Wrist Device Recognize Daily Activities and Detect Falls? *Sensors (Basel).* **16**, (2016).

8. Dijkstra, B., Zijlstra, W., Scherder, E. & Kamsma, Y. Detection of walking periods and number of steps in older adults and patients with Parkinson’s disease: accuracy of a pedometer and an accelerometry-based method. *Age Ageing* **37**, 436–441 (2008).

9. Urbanek, J. K. *et al.* Prediction of sustained harmonic walking in the free-living environment using raw accelerometry data. *Physiol. Meas.* **39**, 02NT02 (2018).

10. Hickey, A., Del Din, S., Rochester, L. & Godfrey, A. Detecting free-living steps and walking bouts: validating an algorithm for macro gait analysis. *Physiol. Meas.* **38**, N1–N15 (2017).

11. Mannini, A., Rosenberger, M., Haskell, W. L., Sabatini, A. M. & Intille, S. S. Activity Recognition in Youth Using Single Accelerometer Placed at Wrist or Ankle. *Med. Sci. Sports Exerc.* **49**, 801–812 (2017).

12. Ellis, K., Kerr, J., Godbole, S., Staudenmayer, J. & Lanckriet, G. Hip and Wrist Accelerometer Algorithms for Free-Living Behavior Classification. *Med. Sci. Sports Exerc.* **48**, 933–940 (2016).

13. Ray, E. L., Sasaki, J. E., Freedson, P. S. & Staudenmayer, J. Physical activity classification with dynamic discriminative methods. *Biometrics* **74**, 1502–1511 (2018).

14. Sasaki, J. E. *et al.* Performance of Activity Classification Algorithms in Free-Living Older Adults. *Med. Sci. Sports Exerc.* **48**, 941–950 (2016).

15. Xiao, L. *et al.* Movement prediction using accelerometers in a human population. *Biometrics* **72**, 513–524 (2016).

16. Zhang, S., Rowlands, A. V, Murray, P. & Hurst, T. L. Physical activity classification using the GENEA wrist-worn accelerometer. *Med. Sci. Sports Exerc.* **44**, 742–748 (2012).
